# Quantifying the degree of bias from using county‐scale data in species distribution modeling: Can increasing sample size or using county‐averaged environmental data reduce distributional overprediction?

**DOI:** 10.1002/ece3.3115

**Published:** 2017-06-28

**Authors:** Steven D. Collins, John C. Abbott, Nancy E. McIntyre

**Affiliations:** ^1^ Johnson, Mirmiran and Thompson Lake Mary FL USA; ^2^ University of Alabama Museums, The University of Alabama Tuscaloosa AL USA; ^3^ Department of Biological Sciences Texas Tech University Lubbock TX USA

**Keywords:** citizen science, distribution modeling, Lepidoptera, maxent, niche modeling, Odonata

## Abstract

Citizen‐science databases have been used to develop species distribution models (SDMs), although many taxa may be only georeferenced to county. It is tacitly assumed that SDMs built from county‐scale data should be less precise than those built with more accurate localities, but the extent of the bias is currently unknown. Our aims in this study were to illustrate the effects of using county‐scale data on the spatial extent and accuracy of SDMs relative to true locality data and to compare potential compensatory methods (including increased sample size and using overall county environmental averages rather than point locality environmental data). To do so, we developed SDMs in maxent with PRISM‐derived BIOCLIM parameters for 283 and 230 species of odonates (dragonflies and damselflies) and butterflies, respectively, for five subsets from the OdonataCentral and Butterflies and Moths of North America citizen‐science databases: (1) a true locality dataset, (2) a corresponding sister dataset of county‐centroid coordinates, (3) a dataset where the average environmental conditions within each county were assigned to each record, (4) a 50/50% mix of true localities and county‐centroid coordinates, and (5) a 50/50% mix of true localities and records assigned the average environmental conditions within each county. These mixtures allowed us to quantify the degree of bias from county‐scale data. Models developed with county centroids overpredicted the extent of suitable habitat by 15% on average compared to true locality models, although larger sample sizes (>100 locality records) reduced this disparity. Assigning county‐averaged environmental conditions did not offer consistent improvement, however. Because county‐level data are of limited value for developing SDMs except for species that are widespread and well collected or that inhabit regions where small, climatically uniform counties predominate, three means of encouraging more accurate georeferencing in citizen‐science databases are provided.

## 1 INTRODUCTION

Species distribution models (SDMs) map the geographic distribution of empirically defined suitable environmental space for species of interest and as such are valuable tools in conservation (Franklin, 2009). The models are only as good as the data used to build them, however, so ensuring data accuracy and precision are crucial for model usefulness (Araújo & Guisan, 2006; Huston, 2002). Biases and imprecision in species locality data have been the subject of several studies that have proposed various approaches in detecting and compensating for such errors (e.g., Beale & Lennon, 2012; Fithian, Elith, Hastie, & Keith, 2014; Mitchell, Monk, & Laurenson, 2016). Problems with the resolution of locality data, however, have not been assessed. Although conceptually related to the problem of data inaccuracy, the issue of using coarse‐scale locality data (e.g., at the scale of county, province, state, or country rather than point‐scale data) imposes a unique set of decisions that must be made (namely, whether to assign a coordinate, such as at a county's centroid, at which to extract background environmental information to build an SDM, or to take an average value over the range of environmental conditions present at the scale [e.g., county] being used). In many cases, coarse‐resolution locality data may be all that are available, such as when using citizen‐science data (Graham, Haines‐Young, & Field, 2015).

Citizen‐science data are increasingly being used around the world to develop species distribution models. Many of these databases record species localities with precise information (e.g., geographic coordinates), but many others use only anthropogenic designations such as country, state, province, or county (e.g., checklists). Within the USA, many citizen‐science databases focus at the level of county for recording species localities (Appleby, 1991; Angelo & Boufford, 2000; Donnelly, 2004a,b,c; Price & Dorcas, 2011; http://www.butterfliesandmoths.org/; http://mothphotographersgroup.msstate.edu/). Given the value of vouchered data collected by citizen scientists that is now being realized and utilized (Fink et al., 2010; Hassall, 2012; Kery, Gardner, & Monnerat, 2010; Schmeller et al., 2009; van Strien, van Swaay, & Termaat, 2013; Sullivan et al., 2009; Szabo, Vesk, Baxter, & Possingham, 2010; Wood, Sullivan, Iliff, Fink, & Kelling, 2011), it is important that the impacts of using only county‐scale species data to examine species distributions be understood. The use of county as an indication of species presence (or absence) is often a convenient resolution for recording and displaying data, or may be a consequence of inaccurate historical data and early distribution cataloguing (Abbott, 2006; Lotts & Naberhaus, 2016). For example, sometimes data are recorded only as a specific county because localities are poorly or imprecisely described (Graham et al., 2007). Addressing imprecision in species localities has been a focus in species distribution modeling (see Graham et al., 2007 and Rocchini et al., 2011 for some types of locational errors, their effects on SDMs, and recommendations of modeling approaches that are relatively insensitive to such errors). With the case of using county‐scale data, the issue is one of the resolution rather than imprecision of locality data. Although this is conceptually related to the problem of data inaccuracy, the issue of using coarse‐resolution data (e.g., at scale of county) imposes a unique set of decisions that must be made regarding how to extract background environmental data to build a distribution model. For county‐scale data that are common in citizen‐science databases, the issue becomes whether to assign a coordinate, such as at a county's centroid, at which to extract background environmental information, or to take an average value over the range of environmental conditions present at the scale (county) being used. It is currently unknown how SDMs are affected by such coarsely scaled county data, and whether averaging approaches or increasing sample size can overcome the inherent limitations in such data.

**Figure 1:**
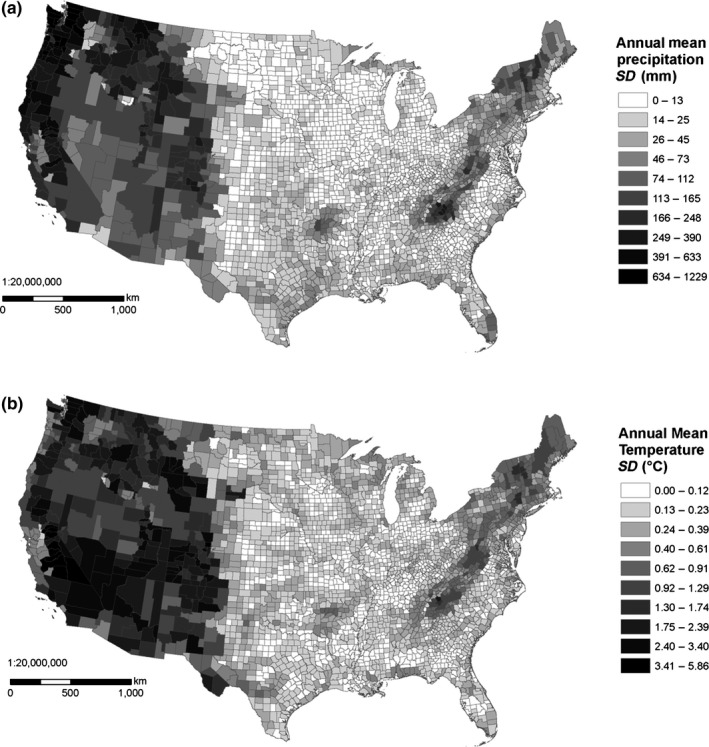
Within‐county standard deviation of (a) annual precipitation and (b) annual mean temperature from the PRISM dataset within the contiguous USA

**Figure 2:**
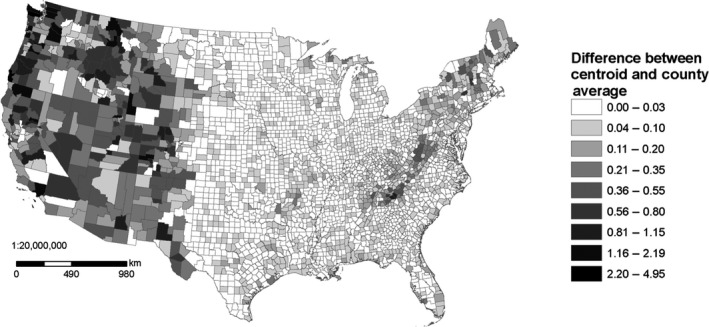
US county centroids tend to be least representative of average county climate in the mountainous and large counties of the western USA. To develop this figure, a principle component analysis (PCA) was performed within the contiguous USA on 19 normalized bioclimatic variables derived from 30‐arcsecond PRISM data (see Methods section). Each component was weighted by the eigenvalue of the PCA to develop an overall climate metric, and the difference between the centroid value and the average value across the county is shown

**Figure 3:**
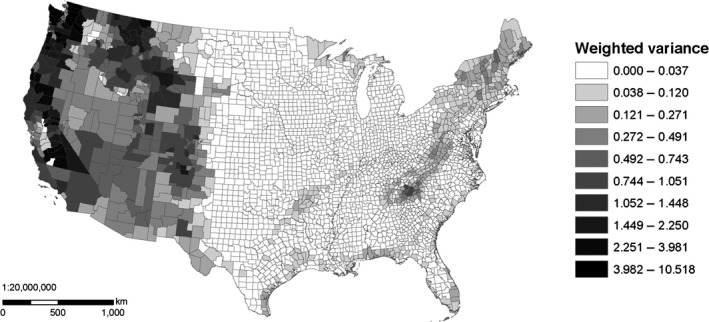
Climatic variance within US counties is highest in the mountainous and large counties of the western USA, though not all large counties have varied climate. Figure generated as in Figure 2, with the variance of each component across each county calculated and weighted by the eigenvalue of the PCA to develop an overall climatic variance metric

The most common environmental data used with SDMs are climatic variables describing the magnitude and seasonality of temperature and precipitation (Pearson & Dawson, 2003). Using county‐scale environmental data may be problematic because associating species‐presence localities with the correct climates is important for SDMs, yet climatic conditions can vary substantially within a US county (Figure 1). Because of the variability of the size of (and thus the climate within) many US counties, species locality data recorded only to the resolution of county might be of limited use for distribution modeling, but this has not previously been examined and so needs to be tested. Previous studies have shown mixed success in parameterizing models at coarse scales and then making predictions at fine scales (Araújo, Thuiller, Williams, & Reginster, 2005; Barbosa, Real, Olivero, & Mario Vargas, 2003; Lloyd & Palmer, 1998; McPherson, Jetz, & Rogers, 2006). Highly uncertain localities such as county‐scale data could be excluded from modeling, but the reduction in sample size could negatively affect model performance (Hernandez, Graham, Master, & Albert, 2006; McPherson, Jetz, & Rogers, 2004), which may be especially true among invertebrates, where as many as 80% of US records are at only the county level for some taxa (Table 1) and are typically recorded with coordinates that represent the geographic center (centroid) of the county. Using the geographic centers of coarse‐scale atlas blocks as localities for distribution models (Lloyd & Palmer, 1998) assumes either that the centroid is representative of suitable climatic conditions within that block or that the entire block contains suitable conditions (McPherson et al., 2006). In some US counties, the centroid may be representative of typical climatic conditions within the county, and these records may thus be highly appropriate for use in distribution modeling. However, in some large or mountainous counties, the centroid may not be representative of the typical climate (Figure 2). This is partly because many large and mountainous counties have different climates contained within their borders (Figure 3). Placing a number of points throughout the county to represent the species occurrence could capture the variability of environmental conditions within the county (Howard, 2006), but it is unlikely that environmental conditions throughout a county are universally suitable for a species, so this approach is likely to increase uncertainty in modeling attempts (McPherson et al., 2006), especially if the point placement scheme is dependent on county size. Another potential solution would be to assign average climatic conditions to county‐data points, though it is unlikely the average is representative of the conditions at the true locality (Huston, 2002). Alternatively, since increased uncertainty may not be inappropriate if it is unknown where precisely a species occurs within a county, a Bayesian modeling approach could be taken that would sample the environmental conditions in a county as covariates (Hanzlicek, Raghavan, Ganta, & Anderson, 2016). Similarly, uncertainty in predictor variables that stems from fine‐scale environmental variation can be addressed with a Bayesian approach (McInerny & Purves, 2011).

**Table 1:** US county centroids and true localities in digital invertebrate databases used in this study

Database	Taxa	Unique county‐only records	Unique true localities^a^	County‐only records without a true locality within the county	True localities where a county record is present	True localities that were a new county record
BAMONA	Butterflies^b^	177,545	36,200	168,536	27,225	5,911
OdonataCentral	Dragonflies and damselflies	94,448	48,130	84,787	20,395	21,551

Database statuses as of November 2012.

a Recorded precision of at least 0.005 decimal degrees (WGS84) ≈555 m.

b Moth records from BAMONA were not analyzed.

**Figure 4:**
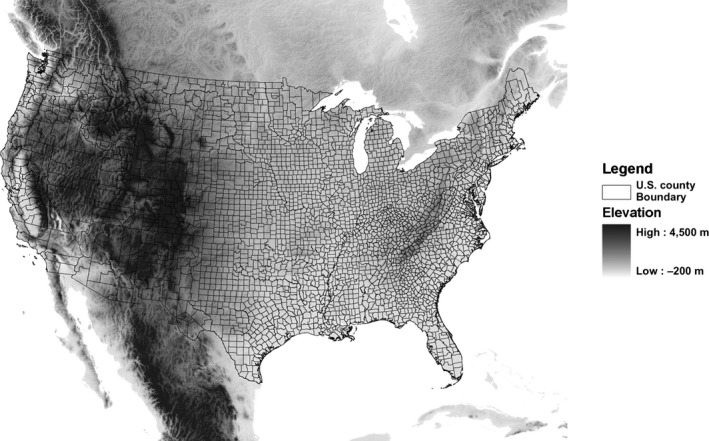
The largest US counties containing the largest elevation ranges tend to be in the western USA

Species distribution models function on the concept that the ecological niche can be defined reasonably with a limited set of environmental variables (e.g., annual precipitation, maximum summer temperature). The environmental conditions at county centroids may be outside the range of suitable conditions for a species, so using centroid data for SDM development may inflate the empirically derived niche, or environmental envelope, and consequently inflate the geographic range of suitable conditions for the species. This study therefore predicts that SDMs developed with US county centroids will overpredict the geographic range of suitable conditions as compared to models developed with true localities and that geographic overprediction will be highest in species that occur in the western USA where large counties include a larger range of environmental conditions compared to smaller eastern US counties (Figure 4).

An ideal test case would be to use a large database of precise species localities from which precise species distribution models can be built and then rescale these localities to a coarser (county) resolution; with these two sets of localities, various approaches to treating background environmental data can then be compared (distribution models built by taking county‐wide environmental averages to those built with county‐centroid environmental values, and combinations of these). Some citizen‐science databases for butterflies and odonates (dragonflies and damselflies) provide such test cases, so our objectives were to determine how species distribution models built using only county‐scale data compared to using true localities. We then asked whether any techniques could be employed that would allow county‐scale data to generate species distribution models that were of comparable accuracy to those built with true localities; specifically, we examined whether using more samples or using county‐scale environmental averages improved model performance.

## 2 MATERIALS AND METHODS

### 2.1 Data sources

Two online citizen‐science species‐presence datasets were used to test our predictions. OdonataCentral (http://www.OdonataCentral.org) and the Butterflies and Moths of North America (BAMONA; http://www.butterfliesandmoths.org) include vouchered records of odonates and butterflies, respectively, from personal and museum collections and photo‐records submitted by professional and citizen scientists that are vetted by experts (Table 1). OdonataCentral also includes the North American Dot‐map Project (DMP) compiled by Nick Donnelly (Donnelly, 2004a,b,c). Though the DMP database is the most comprehensive source of US distributional data contained within OdonataCentral, each record corresponds to a US county centroid for a species, and no other metadata are available. Similarly, the BAMONA database was built from the USGS Northern Prairie Wildlife Research Center database, and no metadata are included with the USGS US county records.

Odonate localities contained within OdonataCentral were obtained in November 2012, and butterfly localities contained within BAMONA were obtained in November 2012. US county records without accurate locality information were excluded from analysis. The OdonataCentral and BAMONA databases were filtered to exclude the following: records outside of the contiguous USA, inaccurately located data points including county centroids and instances where the plotted coordinates did not fall within the user‐entered county, duplicate entries for the same species and locality, records without a photograph or specimen voucher, and records that had been invalidated or not yet vetted by expert reviewers. For species that had more than one validated record within a county, only the first record in the database was selected from each county. This one‐per‐county filter was performed so the sample size for each species would match the sample size of county centroids for each species. Though these datasets may be spatially biased toward population centers or regions with active collectors, for the purpose of this study, it is assumed that the filtered datasets contain an acceptable representation of the distribution of all species. After these filters, only species that contained ten or more records were retained for modeling, resulting in a set of 283 species of odonates and 230 species of butterflies within the USA. Sister datasets were created that contained the coordinates for the county centroid of each record contained in the filtered datasets. To compare if county centroids are more problematic in the western USA versus the eastern USA, species were selected where all filtered records were east or west of the 100th meridian. To reduce the effect of sample size on this comparison, a subset of strictly eastern and western species were selected such that the numbers of records were comparable (Table 2).

**Table 2:** Number of species with all records east or west of the 100th meridian

Database	East of 100th meridian		West of 100th meridian	
	No. of species	No. of records	No. of species	No. of records
OdonataCentral	75	Median = 28, *SD* = 24.85	22	Median = 15, *SD* = 11.01
BAMONA	63	Median = 37, *SD* = 49.18	60	Median = 14.5, *SD* = 5.82
OdonataCentral (subset)	22	Median = 15.5, *SD* = 10.40	22	Median = 15, *SD* = 11.01
BAMONA (subset)	30	Median = 20.5, *SD* = 6.20	30	Median = 19, *SD* = 5.03

### 2.2 Model development

Bioclimatic (bioclim) variables were derived from monthly values of maximum and minimum temperature and precipitation from the PRISM 30‐arcsec climatology normals (1971–2000) gridded dataset (http://www.prism.oregonstate.edu). These 19 metrics are considered more biologically meaningful than the 36 raw monthly values (Busby, 1991). PRISM was chosen because it is more physiographically sensitive than WorldClim and performs better in mountainous regions (Daly et al., 2008). The better performance of PRISM can be attributed to interpolation method, an increased density of available weather stations, and a peer‐review procedure that accounts for local knowledge in the development process (Daly et al., 2008). The bioclim grids and the odonate and butterfly datasets were reprojected to NAD_1983_Albers to avoid the cell‐size bias with latitude present in the native projections of these data.

The 19 bioclim metrics are often correlated with each other, so a principal component analysis (PCA) was performed so that a subset of uncorrelated layers describing climate could be used for model development. Using PCA for this purpose is recommended over using full sets of potentially correlated variables, such as bioclim data (Porfirio et al., 2014). To normalize each bioclim grid, the mean of all cells within the contiguous USA (9,154,303 cells) was subtracted from each cell's value, and the result in each cell was divided by the standard deviation of all cells. This resulted in 19 raster layers, each having an average of 0 and a standard deviation of 1. Principal components were derived from these 19 layers with the principal components tool in ArcGIS 9.3. The first principal component explained 40.6%, the first three components explained 82.6%, and the first seven explained 98.5% of the total variance in the original 19 bioclim grids, so SDMs were developed using the first seven principal component grids as background environmental layers.

Species distribution modeling was performed in maxent v.3.3.3k (Phillips, Dudik, & Schapire, 2004). This modeling package has been shown to be robust compared to other methods (Elith et al., 2006) and has been shown to perform well in the face of spatial errors in training localities (Graham et al., 2007) and reduced sample sizes (Wisz et al., 2008). If using only county records for species‐location data in species distribution modeling, one has to either assume average environmental conditions across the county as a whole unit, or select environmental conditions at a representative point location (such as the county centroid). To examine the consequences of these alternatives, SDMs were generated for each of the 283 focal odonate species and 230 focal butterfly species using five datasets: (1) the true species locality dataset, (2) the corresponding sister dataset of county‐centroid coordinates, (3) a dataset where the average environmental conditions within each county were assigned to each record, (4) a dataset including a 50/50% mix of true localities and county‐centroid coordinates, and (5) a dataset including a 50/50% mix of true localities and records assigned the average environmental conditions within each county. For the two 50/50% mix approaches, the records converted to county‐scale data were selected randomly, but the same selection of true localities and county‐scale data were used in both approaches.

### 2.3 Data analysis

The model outputs from each dataset were compared in terms of areal extent and niche similarity metrics. For the purposes of this project, models developed with true localities were assumed to represent the true distribution of each species. It is known that locality density will be highest in regions with small counties, but this bias should be consistent among all model comparisons. To quantify and compare the areal extent of each modeled range between models developed with each dataset for each species, two thresholds were applied for each species. All grid cells with a predicted value above the threshold were considered suitable, and the number of suitable cells was summed to quantify the areal extent of each distribution. The sensitivity‐equals‐specificity threshold represented the value where positive and negative observations have an equal chance of being predicted correctly, and the minimum‐training‐presence threshold represented the value where a species was predicted to be present at all localities used to train the model (Fielding & Bell, 1997; Freeman & Moisen, 2008). Overprediction was assessed by the ratio of the areal extent of thresholded model output for each dataset compared to the areal extent of the thresholded model output for the true locality models. Comparisons were also made between eastern and western species subsets (Table 2).

The niche similarity of models generated with each dataset was compared for each species using ENMTools (Warren, Glor, & Turelli, 2010). Three statistics were generated: the similarity statistic (I), which is a metric used to test whether models generated from different populations are identical; Schoener's D, which is a metric describing the level of niche overlap; and relative rank (RR), which is an estimate of the probability that a pair of rasters agree in the relative ranking of any two patches of habitat regardless of the suitability values (Warren, Glor, & Turelli, 2008; Warren & Seifert, 2011; Warren et al., 2010). For both I and D, a value of 0 represents no niche overlap, and a value of 1 represents identical niches. For RR, a value of 0 represents disagreement on the relative quality of every habitat patch (cell) pairing, and a value of 1 represents identical relative ranking of all cell pairs. Values of I, D, and RR were compared across models via nonparametric (Wilcoxon signed‐rank) tests.

Area under the curve (AUC) values were also compared between models for each species with Wilcoxon signed‐rank tests. AUC is a threshold‐independent measure of model performance (Hanley & McNeil, 1982), where performance is assessed as the ability of the model to discriminate between species occurrence and absence. Use of this metric is a standard technique for distribution models based on presence‐only data (Guisan & Zimmermann, 2000). AUC values above 0.9 typically describe very good discrimination ability (Swets, 1988).

## 3 RESULTS

Species distribution models for 283 species of odonates and 230 species of butterflies in the continental USA based on true locality data outperformed corresponding SDMs based on county centroids, with a mean AUC reduction of 0.007 for the latter models (Wilcoxon signed‐rank test *p* < 0.00001). The AUC of county‐centroid SDMs was still fairly high (median: 0.915, min: 0.733, max: 0.996). Developing niche models with county data led to overprediction, on average, of the geographic representation of the environmental niche (mean: 15%; median: 3.2%; range: −65% to 789%). The mean predicted areas were larger regardless of how the county‐scale data were treated (Table 3). The majority of butterfly and odonate species (*n* = 288) showed greater than a 1.0% increase in the predicted range when county centroids were substituted for true localities, although 201 species showed a reduction in range by more than 1.0%, and 24 species showed less than 1.0% change (Appendix S1). Considering both butterflies and odonates, the lowest and highest RR metrics for a county‐centroid model compared to a true locality model were 0.737 and 0.988. The lowest and highest RR metrics for a county‐average model compared to a true locality model were 0.751 and 0.996. The lowest metric values were associated with species with few localities. The butterfly and odonate models built from a 50/50% mix of true localities and county centroids significantly outperformed the models built with county centroids as assessed by the I, D, and RR metrics. Specifically, for the butterfly models, the county‐average models outperformed the county‐centroid models as assessed by the I, D, and RR metrics (Wilcoxon signed‐rank test *p* = 0.0179, *p* = 0.0061, and *p* = 0.0311 for I, D, and RR, respectively), and for the odonate models, the county‐centroid models outperformed the county‐average models as assessed by the I, D, and RR metrics (*p* < 0.00001 for all three metrics).

**Table 3:** Mean ratio in predicted area relative to predicted area using true localities

Trial	Equal specificity/sensitivity threshold (%)	Minimum‐training‐presence threshold (%)
BAMONA (*n* = 230)		
County centroid	106.8	110.5
County average	100.6	101.4
50/50 True locality/county‐centroid mix	106.5	109.4
50/50 True locality/county‐average mix	102.8	103.3
OdonataCentral (*n* = 283)		
County centroid	109.4	119.0
County average	100.7	107.9
50/50 True locality/county‐centroid mix	105.7	114.2
50/50 True locality/county‐average mix	103.3	109.3

**Figure 5:**
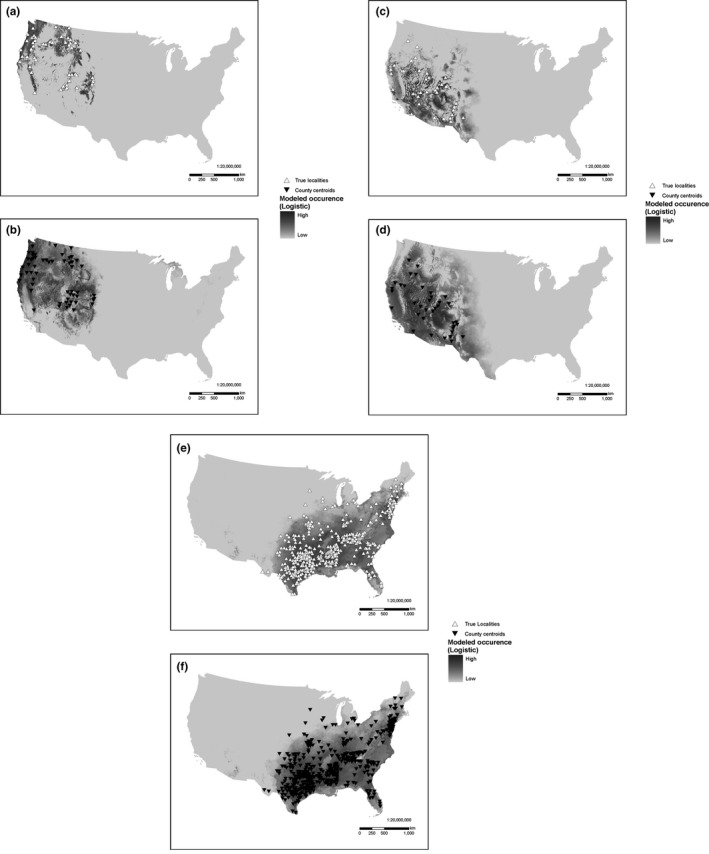
Predicted ranges using true localities and county centroids for some representative odonate taxa: (a) *Somatochlora semicircularis* (*n* = 55) TL, (b) *Somatochlora semicircularis* (*n* = 55) CC, (c) *Erpetogomphus compositus* (*n* = 37) TL, (d) *Erpetogomphus compositus* (*n* = 37) CC, (e) *Ischnura posita* (*n* = 423) TL, (f) *Ischnura posita* (*n* = 423) CC. TL, true localities (white triangles); CC, county centroids (black triangles)

**Figure 6:**
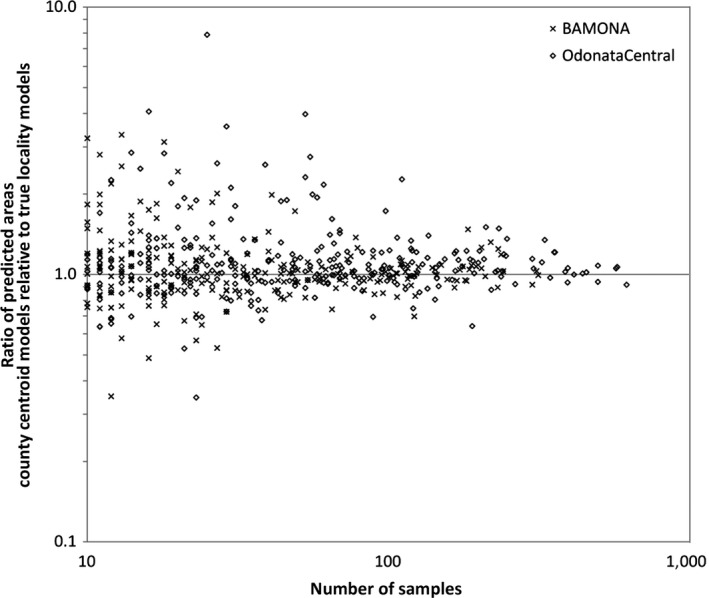
The discrepancy in predicted area using US county centroids relative to true localities is greatest for species with few locality records, using the minimum‐training‐presence threshold for predicted area assuming a default value of prevalence. “x” = data from BAMONA, “o” = data from OdonataCentral

Misrepresentation of the geographic distribution of a species, an effect of modeling with county centroids, was reduced in more widespread species, which had records in many US counties (some examples in Figure 5a–f). The largest predicted area discrepancies were found in species with few records (Figure 6), though not all species with few records showed large discrepancies, and large sample sizes (>100 locality records) reduced the discrepancies. Geographic overprediction tended to be highest in species that had records in counties with high climatic variance, such as western US counties (Figure 3). The median increases in predicted area using county centroids in the western species subsets as indicated in Table 2 were 19.2% and 17.9% for BAMONA and OdonataCentral, respectively. The median increases in predicted area in the eastern species subsets were smaller: −1.7% and 3.0% for BAMONA and OdonataCentral, respectively (*p* = 0.008).

## 4 DISCUSSION

A couple of large citizen‐science datasets were used to test the effects of different approaches in dealing with coarse‐scale data, taking a novel approach: geographic locality data were used to build precise species distribution models and also rescaled the resolution of those same data to a coarser (county) level. With these species localities, distribution models built by taking county‐wide environmental averages were compared to those built with county‐centroid environmental values, and combinations of these (compared to models built with the same data but from specified coordinates). We show that none of these compromises is particularly effective for large, heterogeneous counties of the western USA, although large sample sizes can reduce the possibility of overpredicting species distributions. Using county‐scale data somewhat compromises species distributional predictions, although this effect is more pronounced in the large and environmentally heterogeneous western US counties. Our attempts to compensate for this bias (e.g., by increasing sample size in terms of species locality data, or by comparing models built with county‐averaged environmental data vs. point location [county centroid] environmental data) had mixed success. There was not a consistent performance benefit to using county‐average environmental conditions compared to county centroids. The predicted range sizes of models built with county‐average environmental conditions were closer to models built with true locality data for both butterflies and odonates (Table 3), but comparisons using I, D, and RR metrics failed to reveal any consistent improvements (Table 4). It is thus possible that model performance may be improved by filtering and omitting points from counties with highly variable environmental conditions while keeping those from less variable counties, but this would need to be tested (possibly on a case‐by‐case basis).

**Table 4:** Mean niche similarity metrics compared to true locality models

Trial	I	D	RR
BAMONA (*n* = 230)			
County centroid	0.9829	0.8860	0.9188
County average	0.9870	0.8961	0.9247
50/50 True locality/county‐centroid mix	0.9915	0.9209	0.9439
50/50 True locality/county‐average mix	0.9906	0.9176	0.9349
OdonataCentral (*n* = 283)			
County centroid	0.9837	0.8917	0.9210
County average	0.9802	0.8810	0.9132
50/50 True locality/county‐centroid mix	0.9925	0.9273	0.9475
50/50 True locality/county‐average mix	0.9924	0.9264	0.9459

The similarity statistic I is a metric used to test if models generated from different populations are identical. Schoener's D is a metric describing the level of niche overlap. Relative rank, RR, is an estimate of the probability that a pair of rasters agree in the relative ranking of any two patches of habitat regardless of the suitability values. See text for interpretation.

For widespread species with many county‐scale data points, the risk of inflating the predicted range of a species is small. It is already known that model accuracy declines with reduced sample size, with model performance declining by as much as 19% when using 10 samples instead of 100 (Wisz et al., 2008), although model accuracy may be compromised even further for species with restricted ranges, which could have a corresponding low number of potentially unrepresentative county‐centroid records. It may be possible to use county‐scale data to complement true localities for model development, but the models should be interpreted carefully. Without an independent dataset for comparison, expert opinion on distributions and habitat association may be the best means for determining whether models including county‐scale data are improvements to models developed with only true localities (Burgman, 2005; Perera, Drew, & Johnson, 2012).

From this, we can conclude that high‐resolution SDMs should not be built with coarse‐resolution data. If those are the only such data available, however, using county‐average environmental conditions does not improve model performance (particularly for heterogeneous counties), although boosting sample size of species locality data showed some model improvement. Instead, some alternative approaches may be taken to improve model reliability. For example, background samples could be limited to locations where there are species records, which would control for sampling bias. Furthermore, rather than compare the AUC of models built from different types of data as we did (e.g., compare AUC of true locality model to AUC of county‐centroid model), models could be built with k‐fold partitioning to evaluate all models on the same withheld fold of true locality data. However, withholding a test dataset for comparing the AUC of the true locality and county‐data models would have further reduced the number of species that could be modeled. Moreover, species with few locality records could not be modeled in that manner, which would limit understanding how the different modeling approaches affected species with few records (species with few records could be disproportionately affected if county data were substituted). Additionally, comparing predicted areas and niche similarity indices makes reporting AUC secondary.

There is value to county‐scale data in that it provides at least some evidence of the occurrence of a species, though including it may provide an overly optimistic view of the completeness and extent of data coverage. It is important to understand biases that may be present in citizen‐science databases. Citizen‐science databases are likely biased toward the locations of active users, because there are seldom going to be attempts to randomize localities or search beyond what is convenient for users. The data may suggest some areas to be biodiversity hot spots and others depauperate simply because of differences in effort. For the two databases used in this study, this was a bias in user‐submitted records: contributors are more likely to submit records with actual coordinates if no record existed in a county previously regardless of the precision of that previous record (Table 1). Table 1 shows that US county‐scale records outnumbered true locality records in both databases and that the majority of US county records do not have a true locality record within the same county. In OdonataCentral and BAMONA, 44.8% and 16.3% of true locality records were new county records (records where no accurate or inaccurate record for the county was present in the database), respectively. Database users submit records when they perceive they are contributing new information, and contributing records within a county that already has a record in the database may be perceived as inconsequential. This problem could be addressed in three ways. First, by developing tools that organize data by geographic units other than county, such as watershed, or by providing flight season charts based on existing data, it would be easier for users to identify knowledge gaps in the database. Second, the perceived value of record submission would also increase by presenting the data in other formats. By making record submission easier, users would be more likely to submit more records, including those perceived to be more mundane. This can be accomplished by improving the web submission process or by developing record submission applications for mobile devices (an OdonataCentral mobile app, Dragonfly ID, has been developed, with the ability to include record submission directly from the app coming soon; www.birdseyebirding.com). Both of these improvements could have the added effect of improving record accuracy. Finally, incorporating more user‐centric tools would motivate many contributors. For example, these databases could keep track of user “life‐lists” based on submitted records and list the top contributors by county, state, or country. Though “life‐list” tools would be expected to have only limited scientific value, they would increase the record submission rate. Providing such incentives is important (Wood et al., 2011), because the questions that can be answered with citizen‐science databases are dependent on the number of quality records they contain, and the results of our study indicate that SDMs for species with over 100 county‐centroid records are relatively accurate. Citizen‐science databases should stress the significance of recording specific locality data, because we show that species distribution models based on county‐scale data are inherently flawed.

## CONFLICT OF INTEREST

None declared.
