# Supplementary material for: Quantifying the degree of bias from using county‐scale data in species distribution modeling: Can increasing sample size or using county‐averaged environmental data reduce distributional overprediction?
Source: Ecol Evol. 2017 Jun 28;7(15):6012–22. doi: 10.1002/ece3.3115 (PMC5551104; doi:10.1002/ece3.3115)
Supplement: Supplementary file 1 [file ECE3-7-6012-s001.docx]

**Supporting Material**

**Appendix S1** Change in predicted area within the contiguous United States for each modeled species (n = 230 butterflies, 283 odonates) when county centroids are substituted for true localities. Predicted areas are defined as portion of scene using the minimum training presence threshold.

| Species | Database | No. of Training samples | True Locality Model Predicted Area (Portion of Scene) | Centroid Model Predicted Area (Portion of Scene) | Change in Predicted Area (%) |
| --- | --- | --- | --- | --- | --- |
| *Abaeis nicippe* | BAMONA | 110 | 0.547 | 0.598 | 9.2% |
| *Achalarus lyciades* | BAMONA | 47 | 0.233 | 0.196 | −15.9% |
| *Adelpha californica* | BAMONA | 18 | 0.050 | 0.156 | 213.2% |
| *Aglais milberti* | BAMONA | 65 | 0.837 | 0.621 | −25.8% |
| *Agraulis vanillae* | BAMONA | 166 | 0.541 | 0.484 | −10.6% |
| *Amblyscirtes aesculapius* | BAMONA | 16 | 0.184 | 0.151 | −17.9% |
| *Amblyscirtes hegon* | BAMONA | 40 | 0.310 | 0.378 | 22.1% |
| *Amblyscirtes vialis* | BAMONA | 45 | 0.855 | 0.692 | −19.1% |
| *Anaea andria* | BAMONA | 59 | 0.511 | 0.481 | −5.9% |
| *Anartia jatrophae* | BAMONA | 18 | 0.065 | 0.053 | −19.4% |
| *Anatrytone logan* | BAMONA | 67 | 0.481 | 0.428 | −11.1% |
| *Ancyloxypha numitor* | BAMONA | 126 | 0.672 | 0.717 | 6.8% |
| *Anthanassa texana* | BAMONA | 14 | 0.312 | 0.299 | −4.1% |
| *Anthocharis midea* | BAMONA | 60 | 0.298 | 0.318 | 6.9% |
| *Anthocharis sara* | BAMONA | 11 | 0.038 | 0.055 | 46.3% |
| *Anthocharis stella* | BAMONA | 12 | 0.201 | 0.268 | 33.3% |
| *Apodemia mormo* | BAMONA | 19 | 0.276 | 0.326 | 18.0% |
| *Ascia monuste* | BAMONA | 10 | 0.101 | 0.101 | 0.6% |
| *Asterocampa celtis* | BAMONA | 145 | 0.634 | 0.632 | −0.2% |
| *Asterocampa clyton* | BAMONA | 76 | 0.437 | 0.448 | 2.5% |
| *Atalopedes campestris* | BAMONA | 123 | 0.791 | 0.657 | −16.8% |
| *Atlides halesus* | BAMONA | 54 | 0.664 | 0.634 | −4.6% |
| *Atrytonopsis hianna* | BAMONA | 21 | 0.743 | 0.816 | 9.9% |
| *Autochton cellus* | BAMONA | 14 | 0.445 | 0.424 | −4.7% |
| *Battus philenor* | BAMONA | 157 | 0.737 | 0.706 | −4.1% |
| *Battus polydamas* | BAMONA | 13 | 0.091 | 0.094 | 2.4% |
| *Boloria bellona* | BAMONA | 48 | 0.284 | 0.286 | 0.8% |
| *Boloria selene* | BAMONA | 34 | 0.485 | 0.420 | −13.5% |
| *Brephidium exilis* | BAMONA | 32 | 0.474 | 0.409 | −13.7% |
| *Callophrys augustinus* | BAMONA | 43 | 0.770 | 0.675 | −12.4% |
| *Callophrys eryphon* | BAMONA | 13 | 0.124 | 0.414 | 233.2% |
| *Callophrys gryneus* | BAMONA | 75 | 0.704 | 0.824 | 17.1% |
| *Callophrys henrici* | BAMONA | 44 | 0.643 | 0.648 | 0.8% |
| *Callophrys irus* | BAMONA | 17 | 0.169 | 0.196 | 15.7% |
| *Callophrys johnsoni* | BAMONA | 27 | 0.099 | 0.125 | 26.5% |
| *Callophrys niphon* | BAMONA | 62 | 0.403 | 0.421 | 4.5% |
| *Callophrys perplexa* | BAMONA | 16 | 0.034 | 0.059 | 74.6% |
| *Callophrys sheridanii* | BAMONA | 10 | 0.295 | 0.230 | −22.0% |
| *Callophrys spinetorum* | BAMONA | 14 | 0.563 | 0.671 | 19.0% |
| *Calpodes ethlius* | BAMONA | 14 | 0.279 | 0.300 | 7.6% |
| *Calycopis cecrops* | BAMONA | 116 | 0.392 | 0.414 | 5.6% |
| *Carterocephalus palaemon* | BAMONA | 32 | 0.737 | 0.607 | −17.6% |
| *Celastrina echo* | BAMONA | 29 | 0.220 | 0.265 | 20.3% |
| *Celastrina ladon* | BAMONA | 78 | 0.650 | 0.895 | 37.7% |
| *Celastrina lucia* | BAMONA | 29 | 0.702 | 0.510 | −27.4% |
| *Celastrina neglecta* | BAMONA | 107 | 0.412 | 0.416 | 1.0% |
| *Celastrina serotina* | BAMONA | 21 | 0.047 | 0.041 | −12.9% |
| *Cercyonis oetus* | BAMONA | 11 | 0.158 | 0.442 | 180.6% |
| *Cercyonis pegala* | BAMONA | 114 | 0.638 | 0.653 | 2.4% |
| *Cercyonis sthenele* | BAMONA | 12 | 0.370 | 0.129 | −65.0% |
| *Chlosyne acastus* | BAMONA | 12 | 0.219 | 0.270 | 23.4% |
| *Chlosyne gorgone* | BAMONA | 16 | 0.598 | 0.680 | 13.6% |
| *Chlosyne harrisii* | BAMONA | 26 | 0.081 | 0.150 | 86.3% |
| *Chlosyne lacinia* | BAMONA | 26 | 0.584 | 0.538 | −7.9% |
| *Chlosyne leanira* | BAMONA | 13 | 0.409 | 0.312 | −23.8% |
| *Chlosyne nycteis* | BAMONA | 96 | 0.416 | 0.355 | −14.7% |
| *Chlosyne palla* | BAMONA | 11 | 0.305 | 0.365 | 19.6% |
| *Coenonympha tullia* | BAMONA | 72 | 0.880 | 0.813 | −7.6% |
| *Colias alexandra* | BAMONA | 12 | 0.349 | 0.316 | −9.7% |
| *Colias eurytheme* | BAMONA | 173 | 0.820 | 0.855 | 4.3% |
| *Colias philodice* | BAMONA | 114 | 0.658 | 0.593 | −9.9% |
| *Copaeodes aurantiaca* | BAMONA | 18 | 0.208 | 0.174 | −16.3% |
| *Copaeodes minima* | BAMONA | 20 | 0.132 | 0.115 | −12.5% |
| *Cupido amyntula* | BAMONA | 21 | 0.721 | 0.736 | 2.1% |
| *Cupido comyntas* | BAMONA | 196 | 0.692 | 0.742 | 7.2% |
| *Cyllopsis gemma* | BAMONA | 59 | 0.336 | 0.324 | −3.6% |
| *Danaus gilippus* | BAMONA | 41 | 0.306 | 0.347 | 13.3% |
| *Danaus plexippus* | BAMONA | 237 | 0.833 | 0.990 | 18.8% |
| *Echinargus isola* | BAMONA | 27 | 0.796 | 0.423 | −46.9% |
| *Enodia anthedon* | BAMONA | 111 | 0.393 | 0.444 | 12.8% |
| *Enodia creola* | BAMONA | 18 | 0.166 | 0.142 | −14.2% |
| *Enodia portlandia* | BAMONA | 24 | 0.116 | 0.114 | −1.6% |
| *Epargyreus clarus* | BAMONA | 232 | 0.758 | 0.945 | 24.7% |
| *Erynnis baptisiae* | BAMONA | 102 | 0.372 | 0.391 | 5.3% |
| *Erynnis brizo* | BAMONA | 54 | 0.725 | 0.807 | 11.3% |
| *Erynnis funeralis* | BAMONA | 36 | 0.447 | 0.443 | −1.0% |
| *Erynnis horatius* | BAMONA | 92 | 0.473 | 0.444 | −6.1% |
| *Erynnis icelus* | BAMONA | 40 | 0.536 | 0.775 | 44.5% |
| *Erynnis juvenalis* | BAMONA | 89 | 0.412 | 0.480 | 16.5% |
| *Erynnis persius* | BAMONA | 13 | 0.236 | 0.600 | 153.9% |
| *Erynnis tristis* | BAMONA | 11 | 0.066 | 0.074 | 12.9% |
| *Erynnis zarucco* | BAMONA | 14 | 0.099 | 0.087 | −11.8% |
| *Euchloe ausonides* | BAMONA | 15 | 0.148 | 0.277 | 87.6% |
| *Euphilotes battoides* | BAMONA | 11 | 0.247 | 0.207 | −16.5% |
| *Euphilotes enoptes* | BAMONA | 12 | 0.024 | 0.053 | 118.7% |
| *Euphydryas anicia* | BAMONA | 17 | 0.403 | 0.263 | −34.8% |
| *Euphydryas chalcedona* | BAMONA | 20 | 0.069 | 0.169 | 142.8% |
| *Euphydryas colon* | BAMONA | 10 | 0.158 | 0.235 | 48.9% |
| *Euphydryas editha* | BAMONA | 22 | 0.161 | 0.287 | 78.4% |
| *Euphydryas phaeton* | BAMONA | 45 | 0.240 | 0.262 | 8.9% |
| *Euphyes bimacula* | BAMONA | 10 | 0.711 | 0.537 | −24.5% |
| *Euphyes conspicua* | BAMONA | 14 | 0.147 | 0.244 | 66.2% |
| *Euphyes dion* | BAMONA | 24 | 0.466 | 0.433 | −7.2% |
| *Euphyes vestris* | BAMONA | 107 | 0.976 | 0.804 | −17.6% |
| *Euptoieta claudia* | BAMONA | 180 | 0.716 | 0.682 | −4.7% |
| *Eurytides marcellus* | BAMONA | 116 | 0.548 | 0.527 | −3.7% |
| *Feniseca tarquinius* | BAMONA | 59 | 0.345 | 0.344 | −0.3% |
| *Glaucopsyche lygdamus* | BAMONA | 49 | 0.827 | 0.778 | −5.9% |
| *Glaucopsyche piasus* | BAMONA | 13 | 0.267 | 0.305 | 14.3% |
| *Heliconius charithonia* | BAMONA | 19 | 0.305 | 0.357 | 17.3% |
| *Hemiargus ceraunus* | BAMONA | 27 | 0.481 | 0.486 | 1.0% |
| *Hermeuptychia sosybius* | BAMONA | 72 | 0.297 | 0.291 | −2.0% |
| *Hesperia colorado* | BAMONA | 18 | 0.209 | 0.196 | −5.9% |
| *Hesperia juba* | BAMONA | 11 | 0.259 | 0.274 | 5.9% |
| *Hesperia leonardus* | BAMONA | 34 | 0.316 | 0.317 | 0.3% |
| *Hesperia metea* | BAMONA | 22 | 0.211 | 0.220 | 3.8% |
| *Hesperia sassacus* | BAMONA | 25 | 0.117 | 0.118 | 0.4% |
| *Hylephila phyleus* | BAMONA | 186 | 0.730 | 0.763 | 4.5% |
| *Junonia coenia* | BAMONA | 315 | 0.934 | 0.930 | −0.5% |
| *Leptotes cassius* | BAMONA | 13 | 0.285 | 0.324 | 13.9% |
| *Leptotes marina* | BAMONA | 23 | 0.794 | 0.564 | −29.0% |
| *Lerema accius* | BAMONA | 83 | 0.391 | 0.371 | −5.2% |
| *Lerodea eufala* | BAMONA | 50 | 0.378 | 0.393 | 4.0% |
| *Libytheana carinenta* | BAMONA | 116 | 0.598 | 0.651 | 8.9% |
| *Limenitis archippus* | BAMONA | 140 | 0.778 | 0.825 | 6.1% |
| *Limenitis arthemis* | BAMONA | 95 | 0.423 | 0.423 | 0.0% |
| *Limenitis arthemis arthemis* | BAMONA | 39 | 0.380 | 0.281 | −26.1% |
| *Limenitis arthemis astyanax* | BAMONA | 150 | 0.465 | 0.505 | 8.5% |
| *Limenitis lorquini* | BAMONA | 29 | 0.222 | 0.207 | −7.1% |
| *Limenitis weidemeyerii* | BAMONA | 15 | 0.145 | 0.211 | 45.4% |
| *Lycaena cupreus* | BAMONA | 10 | 0.284 | 0.259 | −8.8% |
| *Lycaena epixanthe* | BAMONA | 14 | 0.149 | 0.179 | 20.6% |
| *Lycaena helloides* | BAMONA | 24 | 0.334 | 0.386 | 15.7% |
| *Lycaena heteronea* | BAMONA | 11 | 0.237 | 0.254 | 7.1% |
| *Lycaena hyllus* | BAMONA | 44 | 0.669 | 0.549 | −17.9% |
| *Lycaena phlaeas* | BAMONA | 79 | 0.692 | 0.871 | 25.9% |
| *Megathymus yuccae* | BAMONA | 13 | 0.454 | 0.587 | 29.4% |
| *Megisto cymela* | BAMONA | 119 | 0.404 | 0.398 | −1.3% |
| *Nastra lherminier* | BAMONA | 24 | 0.263 | 0.294 | 11.9% |
| *Nathalis iole* | BAMONA | 98 | 0.888 | 0.910 | 2.5% |
| *Neophasia menapia* | BAMONA | 16 | 0.223 | 0.170 | −23.6% |
| *Nymphalis antiopa* | BAMONA | 210 | 0.933 | 0.919 | −1.5% |
| *Nymphalis californica* | BAMONA | 16 | 0.728 | 0.632 | −13.1% |
| *Nymphalis vaualbum* | BAMONA | 28 | 0.567 | 0.468 | −17.5% |
| *Ochlodes sylvanoides* | BAMONA | 20 | 0.493 | 0.518 | 5.0% |
| *Oeneis chryxus* | BAMONA | 10 | 0.080 | 0.258 | 223.3% |
| *Panoquina ocola* | BAMONA | 50 | 0.300 | 0.281 | −6.3% |
| *Papilio canadensis* | BAMONA | 29 | 0.476 | 0.416 | −12.5% |
| *Papilio cresphontes* | BAMONA | 182 | 0.740 | 0.701 | −5.3% |
| *Papilio eurymedon* | BAMONA | 26 | 0.254 | 0.222 | −12.4% |
| *Papilio glaucus* | BAMONA | 311 | 0.541 | 0.558 | 3.1% |
| *Papilio indra* | BAMONA | 16 | 0.322 | 0.287 | −11.0% |
| *Papilio multicaudata* | BAMONA | 33 | 0.607 | 0.649 | 6.9% |
| *Papilio palamedes* | BAMONA | 31 | 0.139 | 0.146 | 4.8% |
| *Papilio polyxenes* | BAMONA | 241 | 0.758 | 0.780 | 2.9% |
| *Papilio rutulus* | BAMONA | 36 | 0.306 | 0.416 | 35.6% |
| *Papilio troilus* | BAMONA | 169 | 0.470 | 0.446 | −5.1% |
| *Papilio zelicaon* | BAMONA | 23 | 0.357 | 0.401 | 12.5% |
| *Parnassius clodius* | BAMONA | 18 | 0.144 | 0.184 | 28.1% |
| *Parnassius smintheus* | BAMONA | 10 | 0.136 | 0.249 | 82.8% |
| *Parrhasius m album* | BAMONA | 39 | 0.385 | 0.390 | 1.4% |
| *Philotes sonorensis* | BAMONA | 11 | 0.034 | 0.062 | 82.6% |
| *Phoebis philea* | BAMONA | 11 | 0.237 | 0.177 | −25.3% |
| *Phoebis sennae* | BAMONA | 122 | 0.965 | 0.673 | −30.2% |
| *Pholisora catullus* | BAMONA | 68 | 0.950 | 0.957 | 0.7% |
| *Phyciodes cocyta* | BAMONA | 42 | 0.547 | 0.476 | −12.8% |
| *Phyciodes mylitta* | BAMONA | 21 | 0.284 | 0.278 | −2.1% |
| *Phyciodes phaon* | BAMONA | 34 | 0.254 | 0.302 | 19.1% |
| *Phyciodes pulchella* | BAMONA | 23 | 0.658 | 0.373 | −43.3% |
| *Phyciodes tharos* | BAMONA | 231 | 0.817 | 0.731 | −10.5% |
| *Pieris marginalis* | BAMONA | 17 | 0.229 | 0.373 | 62.7% |
| *Pieris oleracea* | BAMONA | 16 | 0.106 | 0.101 | −4.4% |
| *Pieris rapae* | BAMONA | 177 | 0.865 | 0.928 | 7.3% |
| *Pieris virginiensis* | BAMONA | 33 | 0.246 | 0.251 | 2.0% |
| *Plebejus acmon* | BAMONA | 18 | 0.198 | 0.273 | 37.8% |
| *Plebejus icarioides* | BAMONA | 26 | 0.229 | 0.315 | 37.6% |
| *Plebejus lupini* | BAMONA | 19 | 0.359 | 0.329 | −8.5% |
| *Plebejus melissa* | BAMONA | 23 | 0.545 | 0.620 | 13.7% |
| *Plebejus saepiolus* | BAMONA | 13 | 0.350 | 0.202 | −42.2% |
| *Poanes hobomok* | BAMONA | 83 | 0.739 | 0.646 | −12.5% |
| *Poanes massasoit* | BAMONA | 12 | 0.127 | 0.125 | −1.5% |
| *Poanes viator* | BAMONA | 17 | 0.255 | 0.230 | −9.7% |
| *Poanes zabulon* | BAMONA | 122 | 0.354 | 0.350 | −1.0% |
| *Polites mystic* | BAMONA | 31 | 0.759 | 0.661 | −12.9% |
| *Polites origenes* | BAMONA | 47 | 0.342 | 0.346 | 1.3% |
| *Polites peckius* | BAMONA | 117 | 0.706 | 0.698 | −1.1% |
| *Polites themistocles* | BAMONA | 94 | 0.514 | 0.489 | −4.9% |
| *Polites vibex* | BAMONA | 29 | 0.716 | 0.623 | −13.0% |
| *Polygonia comma* | BAMONA | 124 | 0.430 | 0.435 | 1.2% |
| *Polygonia faunus* | BAMONA | 27 | 0.322 | 0.647 | 100.9% |
| *Polygonia gracilis* | BAMONA | 24 | 0.561 | 0.709 | 26.5% |
| *Polygonia interrogationis* | BAMONA | 219 | 0.573 | 0.758 | 32.2% |
| *Polygonia progne* | BAMONA | 41 | 0.264 | 0.524 | 98.6% |
| *Polygonia satyrus* | BAMONA | 19 | 0.252 | 0.324 | 28.5% |
| *Pompeius verna* | BAMONA | 69 | 0.372 | 0.356 | −4.3% |
| *Pontia occidentalis* | BAMONA | 16 | 0.299 | 0.255 | −14.7% |
| *Pontia protodice* | BAMONA | 106 | 0.865 | 0.876 | 1.3% |
| *Pontia sisymbrii* | BAMONA | 11 | 0.343 | 0.421 | 22.6% |
| *Pyrgus communis* | BAMONA | 119 | 0.988 | 0.900 | −8.9% |
| *Pyrgus oileus* | BAMONA | 37 | 0.104 | 0.099 | −5.1% |
| *Pyrisitia lisa* | BAMONA | 87 | 0.538 | 0.520 | −3.4% |
| *Satyrium acadica* | BAMONA | 23 | 0.187 | 0.162 | −13.6% |
| *Satyrium calanus* | BAMONA | 90 | 0.697 | 0.588 | −15.6% |
| *Satyrium californica* | BAMONA | 10 | 0.105 | 0.165 | 57.0% |
| *Satyrium caryaevorus* | BAMONA | 19 | 0.241 | 0.266 | 10.4% |
| *Satyrium edwardsii* | BAMONA | 20 | 0.419 | 0.427 | 1.9% |
| *Satyrium favonius* | BAMONA | 16 | 0.380 | 0.468 | 23.1% |
| *Satyrium favonius ontario* | BAMONA | 14 | 0.294 | 0.279 | −5.0% |
| *Satyrium liparops* | BAMONA | 43 | 0.632 | 0.601 | −4.8% |
| *Satyrium saepium* | BAMONA | 17 | 0.155 | 0.285 | 84.0% |
| *Satyrium sylvinus* | BAMONA | 12 | 0.176 | 0.132 | −24.8% |
| *Satyrium titus* | BAMONA | 55 | 0.722 | 0.750 | 3.9% |
| *Satyrodes appalachia* | BAMONA | 44 | 0.390 | 0.416 | 6.6% |
| *Satyrodes eurydice* | BAMONA | 33 | 0.162 | 0.159 | −1.7% |
| *Speyeria aphrodite* | BAMONA | 49 | 0.305 | 0.526 | 72.7% |
| *Speyeria atlantis* | BAMONA | 24 | 0.431 | 0.279 | −35.2% |
| *Speyeria callippe* | BAMONA | 16 | 0.096 | 0.112 | 16.8% |
| *Speyeria coronis* | BAMONA | 11 | 0.301 | 0.366 | 21.6% |
| *Speyeria cybele* | BAMONA | 184 | 0.558 | 0.824 | 47.7% |
| *Speyeria diana* | BAMONA | 21 | 0.353 | 0.329 | −6.6% |
| *Speyeria hesperis* | BAMONA | 14 | 0.206 | 0.274 | 33.4% |
| *Speyeria hydaspe* | BAMONA | 12 | 0.144 | 0.124 | −14.3% |
| *Speyeria idalia* | BAMONA | 18 | 0.148 | 0.113 | −23.3% |
| *Speyeria mormonia* | BAMONA | 10 | 0.188 | 0.202 | 7.0% |
| *Speyeria zerene* | BAMONA | 11 | 0.201 | 0.169 | −15.8% |
| *Staphylus hayhurstii* | BAMONA | 25 | 0.244 | 0.232 | −4.7% |
| *Strymon melinus* | BAMONA | 189 | 0.896 | 0.969 | 8.2% |
| *Thorybes bathyllus* | BAMONA | 45 | 0.298 | 0.280 | −6.0% |
| *Thorybes pylades* | BAMONA | 73 | 0.747 | 0.851 | 13.9% |
| *Thymelicus lineola* | BAMONA | 53 | 0.584 | 0.479 | −18.0% |
| *Urbanus dorantes* | BAMONA | 11 | 0.176 | 0.157 | −11.1% |
| *Urbanus proteus* | BAMONA | 100 | 0.313 | 0.305 | −2.4% |
| *Vanessa annabella* | BAMONA | 21 | 0.577 | 0.387 | −32.9% |
| *Vanessa atalanta* | BAMONA | 301 | 0.800 | 0.733 | −8.4% |
| *Vanessa cardui* | BAMONA | 204 | 0.890 | 0.940 | 5.6% |
| *Vanessa virginiensis* | BAMONA | 204 | 0.723 | 0.915 | 26.4% |
| *Wallengrenia egeremet* | BAMONA | 57 | 0.382 | 0.365 | −4.2% |
| *Wallengrenia otho* | BAMONA | 36 | 0.299 | 0.309 | 3.4% |
| *Zerene cesonia* | BAMONA | 25 | 0.614 | 0.764 | 24.4% |
| *Zerene eurydice* | BAMONA | 11 | 0.025 | 0.050 | 99.2% |
| *Acanthagrion quadratum* | OdonataCentral | 11 | 0.022 | 0.037 | 70.2% |
| *Aeshna canadensis* | OdonataCentral | 56 | 0.359 | 0.716 | 99.3% |
| *Aeshna clepsydra* | OdonataCentral | 15 | 0.253 | 0.303 | 19.4% |
| *Aeshna constricta* | OdonataCentral | 86 | 0.609 | 0.638 | 4.7% |
| *Aeshna eremita* | OdonataCentral | 30 | 0.277 | 0.446 | 61.1% |
| *Aeshna interrupta* | OdonataCentral | 122 | 0.590 | 0.580 | −1.7% |
| *Aeshna juncea* | OdonataCentral | 25 | 0.067 | 0.532 | 689.0% |
| *Aeshna palmata* | OdonataCentral | 120 | 0.444 | 0.494 | 11.3% |
| *Aeshna sitchensis* | OdonataCentral | 16 | 0.084 | 0.340 | 307.2% |
| *Aeshna subarctica* | OdonataCentral | 15 | 0.175 | 0.193 | 10.5% |
| *Aeshna tuberculifera* | OdonataCentral | 30 | 0.377 | 0.796 | 111.2% |
| *Aeshna umbrosa* | OdonataCentral | 144 | 0.763 | 0.810 | 6.1% |
| *Aeshna verticalis* | OdonataCentral | 18 | 0.203 | 0.203 | −0.2% |
| *Aeshna walkeri* | OdonataCentral | 14 | 0.153 | 0.107 | −30.3% |
| *Amphiagrion abbreviatum* | OdonataCentral | 59 | 0.417 | 0.467 | 12.2% |
| *Amphiagrion saucium* | OdonataCentral | 26 | 0.315 | 0.326 | 3.6% |
| *Anax junius* | OdonataCentral | 455 | 0.977 | 0.997 | 2.0% |
| *Anax longipes* | OdonataCentral | 63 | 0.569 | 0.525 | −7.6% |
| *Anax walsinghami* | OdonataCentral | 18 | 0.285 | 0.295 | 3.4% |
| *Aphylla angustifolia* | OdonataCentral | 30 | 0.082 | 0.094 | 14.3% |
| *Aphylla williamsoni* | OdonataCentral | 72 | 0.174 | 0.173 | −0.6% |
| *Archilestes californicus* | OdonataCentral | 31 | 0.116 | 0.127 | 9.9% |
| *Archilestes grandis* | OdonataCentral | 120 | 0.795 | 0.977 | 22.9% |
| *Argia agrioides* | OdonataCentral | 16 | 0.140 | 0.189 | 34.5% |
| *Argia alberta* | OdonataCentral | 69 | 0.499 | 0.506 | 1.4% |
| *Argia apicalis* | OdonataCentral | 356 | 0.684 | 0.830 | 21.3% |
| *Argia bipunctulata* | OdonataCentral | 30 | 0.317 | 0.253 | −20.1% |
| *Argia emma* | OdonataCentral | 67 | 0.381 | 0.409 | 7.5% |
| *Argia fumipennis* | OdonataCentral | 264 | 0.782 | 0.721 | −7.9% |
| *Argia hinei* | OdonataCentral | 17 | 0.164 | 0.224 | 36.5% |
| *Argia immunda* | OdonataCentral | 68 | 0.659 | 0.784 | 18.9% |
| *Argia leonorae* | OdonataCentral | 20 | 0.105 | 0.158 | 50.0% |
| *Argia lugens* | OdonataCentral | 37 | 0.296 | 0.238 | −19.6% |
| *Argia moesta* | OdonataCentral | 331 | 0.662 | 0.893 | 34.8% |
| *Argia nahuana* | OdonataCentral | 89 | 0.455 | 0.454 | −0.2% |
| *Argia plana* | OdonataCentral | 111 | 0.410 | 0.931 | 127.2% |
| *Argia sedula* | OdonataCentral | 211 | 0.568 | 0.855 | 50.6% |
| *Argia tibialis* | OdonataCentral | 182 | 0.412 | 0.505 | 22.7% |
| *Argia translata* | OdonataCentral | 119 | 0.404 | 0.506 | 25.1% |
| *Argia vivida* | OdonataCentral | 69 | 0.434 | 0.636 | 46.6% |
| *Arigomphus cornutus* | OdonataCentral | 30 | 0.239 | 0.256 | 7.4% |
| *Arigomphus furcifer* | OdonataCentral | 17 | 0.148 | 0.148 | 0.1% |
| *Arigomphus lentulus* | OdonataCentral | 21 | 0.186 | 0.181 | −2.6% |
| *Arigomphus maxwelli* | OdonataCentral | 22 | 0.076 | 0.073 | −4.2% |
| *Arigomphus pallidus* | OdonataCentral | 10 | 0.043 | 0.051 | 19.3% |
| *Arigomphus submedianus* | OdonataCentral | 104 | 0.344 | 0.333 | −3.0% |
| *Arigomphus villosipes* | OdonataCentral | 65 | 0.207 | 0.220 | 6.4% |
| *Basiaeschna janata* | OdonataCentral | 86 | 0.408 | 0.444 | 9.0% |
| *Boyeria grafiana* | OdonataCentral | 12 | 0.149 | 0.158 | 6.0% |
| *Boyeria vinosa* | OdonataCentral | 48 | 0.372 | 0.389 | 4.7% |
| *Brachymesia furcata* | OdonataCentral | 39 | 0.289 | 0.270 | −6.7% |
| *Brachymesia gravida* | OdonataCentral | 135 | 0.404 | 0.358 | −11.4% |
| *Brachymesia herbida* | OdonataCentral | 11 | 0.093 | 0.098 | 5.3% |
| *Brechmorhoga mendax* | OdonataCentral | 55 | 0.365 | 0.395 | 8.1% |
| *Calopteryx aequabilis* | OdonataCentral | 77 | 0.677 | 0.699 | 3.3% |
| *Calopteryx angustipennis* | OdonataCentral | 16 | 0.127 | 0.136 | 6.7% |
| *Calopteryx dimidiata* | OdonataCentral | 31 | 0.179 | 0.178 | −0.8% |
| *Calopteryx maculata* | OdonataCentral | 345 | 0.611 | 0.595 | −2.6% |
| *Cannaphila insularis* | OdonataCentral | 11 | 0.126 | 0.080 | −36.1% |
| *Celithemis amanda* | OdonataCentral | 13 | 0.129 | 0.133 | 2.6% |
| *Celithemis bertha* | OdonataCentral | 14 | 0.117 | 0.116 | −0.5% |
| *Celithemis elisa* | OdonataCentral | 201 | 0.574 | 0.704 | 22.7% |
| *Celithemis eponina* | OdonataCentral | 358 | 0.667 | 0.808 | 21.2% |
| *Celithemis fasciata* | OdonataCentral | 147 | 0.413 | 0.373 | −9.5% |
| *Celithemis martha* | OdonataCentral | 14 | 0.026 | 0.073 | 185.5% |
| *Celithemis ornata* | OdonataCentral | 21 | 0.175 | 0.093 | −47.1% |
| *Celithemis verna* | OdonataCentral | 17 | 0.168 | 0.211 | 25.5% |
| *Chromagrion conditum* | OdonataCentral | 45 | 0.262 | 0.273 | 4.1% |
| *Coenagrion resolutum* | OdonataCentral | 44 | 0.319 | 0.600 | 88.0% |
| *Cordulegaster bilineata* | OdonataCentral | 17 | 0.217 | 0.184 | −14.8% |
| *Cordulegaster diastatops* | OdonataCentral | 23 | 0.066 | 0.091 | 36.9% |
| *Cordulegaster dorsalis* | OdonataCentral | 35 | 0.293 | 0.271 | −7.3% |
| *Cordulegaster erronea* | OdonataCentral | 19 | 0.135 | 0.296 | 119.8% |
| *Cordulegaster maculata* | OdonataCentral | 41 | 0.446 | 0.419 | −6.1% |
| *Cordulegaster obliqua* | OdonataCentral | 64 | 0.479 | 0.543 | 13.2% |
| *Cordulia shurtleffii* | OdonataCentral | 58 | 0.301 | 0.584 | 94.1% |
| *Coryphaeschna adnexa* | OdonataCentral | 12 | 0.101 | 0.069 | −31.2% |
| *Coryphaeschna ingens* | OdonataCentral | 27 | 0.140 | 0.152 | 9.0% |
| *Crocothemis servilia* | OdonataCentral | 12 | 0.012 | 0.013 | 6.7% |
| *Didymops transversa* | OdonataCentral | 95 | 0.335 | 0.351 | 4.7% |
| *Dorocordulia libera* | OdonataCentral | 27 | 0.173 | 0.166 | −3.9% |
| *Dromogomphus spinosus* | OdonataCentral | 121 | 0.627 | 0.469 | −25.2% |
| *Dromogomphus spoliatus* | OdonataCentral | 154 | 0.404 | 0.462 | 14.3% |
| *Dythemis fugax* | OdonataCentral | 95 | 0.197 | 0.238 | 20.9% |
| *Dythemis nigrescens* | OdonataCentral | 34 | 0.108 | 0.103 | −4.0% |
| *Dythemis velox* | OdonataCentral | 124 | 0.395 | 0.480 | 21.3% |
| *Enallagma anna* | OdonataCentral | 69 | 0.684 | 0.662 | −3.3% |
| *Enallagma annexum* | OdonataCentral | 107 | 0.518 | 0.713 | 37.5% |
| *Enallagma antennatum* | OdonataCentral | 101 | 0.482 | 0.595 | 23.3% |
| *Enallagma aspersum* | OdonataCentral | 105 | 0.552 | 0.517 | −6.3% |
| *Enallagma basidens* | OdonataCentral | 248 | 0.597 | 0.812 | 36.1% |
| *Enallagma boreale* | OdonataCentral | 80 | 0.672 | 0.626 | −6.8% |
| *Enallagma carunculatum* | OdonataCentral | 166 | 0.712 | 0.855 | 20.1% |
| *Enallagma civile* | OdonataCentral | 442 | 0.898 | 0.908 | 1.0% |
| *Enallagma clausum* | OdonataCentral | 22 | 0.464 | 0.419 | −9.7% |
| *Enallagma concisum* | OdonataCentral | 11 | 0.050 | 0.055 | 10.9% |
| *Enallagma daeckii* | OdonataCentral | 22 | 0.167 | 0.156 | −6.6% |
| *Enallagma davisi* | OdonataCentral | 10 | 0.076 | 0.067 | −11.9% |
| *Enallagma divagans* | OdonataCentral | 65 | 0.357 | 0.356 | −0.3% |
| *Enallagma doubledayi* | OdonataCentral | 18 | 0.154 | 0.130 | −15.7% |
| *Enallagma dubium* | OdonataCentral | 23 | 0.167 | 0.161 | −3.6% |
| *Enallagma durum* | OdonataCentral | 28 | 0.275 | 0.279 | 1.5% |
| *Enallagma ebrium* | OdonataCentral | 40 | 0.373 | 0.422 | 13.3% |
| *Enallagma exsulans* | OdonataCentral | 190 | 0.846 | 0.543 | −35.8% |
| *Enallagma geminatum* | OdonataCentral | 145 | 0.515 | 0.499 | −3.1% |
| *Enallagma hageni* | OdonataCentral | 97 | 0.402 | 0.434 | 8.1% |
| *Enallagma novaehispaniae* | OdonataCentral | 16 | 0.017 | 0.019 | 10.1% |
| *Enallagma praevarum* | OdonataCentral | 64 | 0.456 | 0.642 | 41.0% |
| *Enallagma signatum* | OdonataCentral | 242 | 0.696 | 0.830 | 19.2% |
| *Enallagma traviatum* | OdonataCentral | 90 | 0.387 | 0.394 | 1.7% |
| *Enallagma vesperum* | OdonataCentral | 70 | 0.472 | 0.499 | 5.8% |
| *Enallagma weewa* | OdonataCentral | 10 | 0.089 | 0.092 | 2.8% |
| *Epiaeschna heros* | OdonataCentral | 125 | 0.391 | 0.400 | 2.1% |
| *Epitheca canis* | OdonataCentral | 23 | 0.258 | 0.273 | 5.9% |
| *Epitheca costalis* | OdonataCentral | 38 | 0.241 | 0.228 | −5.4% |
| *Epitheca cynosura* | OdonataCentral | 130 | 0.459 | 0.501 | 9.2% |
| *Epitheca petechialis* | OdonataCentral | 58 | 0.477 | 0.585 | 22.7% |
| *Epitheca princeps* | OdonataCentral | 193 | 0.514 | 0.520 | 1.2% |
| *Epitheca semiaquea* | OdonataCentral | 44 | 0.284 | 0.375 | 31.9% |
| *Epitheca spinigera* | OdonataCentral | 24 | 0.603 | 0.419 | −30.5% |
| *Erpetogomphus compositus* | OdonataCentral | 37 | 0.376 | 0.276 | −26.5% |
| *Erpetogomphus designatus* | OdonataCentral | 89 | 0.661 | 0.460 | −30.5% |
| *Erpetogomphus lampropeltis* | OdonataCentral | 13 | 0.219 | 0.186 | −14.9% |
| *Erythemis collocata* | OdonataCentral | 82 | 0.373 | 0.453 | 21.4% |
| *Erythemis plebeja* | OdonataCentral | 22 | 0.054 | 0.068 | 26.1% |
| *Erythemis simplicicollis* | OdonataCentral | 576 | 0.725 | 0.774 | 6.8% |
| *Erythemis vesiculosa* | OdonataCentral | 51 | 0.351 | 0.406 | 15.7% |
| *Erythrodiplax berenice* | OdonataCentral | 43 | 0.820 | 0.699 | −14.7% |
| *Erythrodiplax minuscula* | OdonataCentral | 66 | 0.269 | 0.267 | −0.6% |
| *Erythrodiplax umbrata* | OdonataCentral | 66 | 0.317 | 0.414 | 30.8% |
| *Gomphaeschna antilope* | OdonataCentral | 16 | 0.422 | 0.439 | 3.9% |
| *Gomphaeschna furcillata* | OdonataCentral | 48 | 0.378 | 0.451 | 19.2% |
| *Gomphus dilatatus* | OdonataCentral | 19 | 0.164 | 0.140 | −14.5% |
| *Gomphus externus* | OdonataCentral | 96 | 0.591 | 0.620 | 4.9% |
| *Gomphus fraternus* | OdonataCentral | 19 | 0.276 | 0.277 | 0.7% |
| *Gomphus hybridus* | OdonataCentral | 26 | 0.128 | 0.151 | 18.4% |
| *Gomphus vastus* | OdonataCentral | 61 | 0.519 | 0.484 | −6.8% |
| *Hagenius brevistylus* | OdonataCentral | 112 | 0.493 | 0.527 | 6.9% |
| *Helocordulia selysii* | OdonataCentral | 21 | 0.110 | 0.094 | −14.9% |
| *Helocordulia uhleri* | OdonataCentral | 20 | 0.168 | 0.165 | −1.4% |
| *Hesperagrion heterodoxum* | OdonataCentral | 15 | 0.077 | 0.065 | −15.9% |
| *Hetaerina americana* | OdonataCentral | 324 | 0.784 | 0.872 | 11.2% |
| *Hetaerina titia* | OdonataCentral | 74 | 0.389 | 0.432 | 11.1% |
| *Hylogomphus apomyius* | OdonataCentral | 14 | 0.08 | 0.125 | 55.60% |
| *Hylogomphus viridifrons* | OdonataCentral | 10 | 0.265 | 0.236 | -10.7% |
| *Ischnura barberi* | OdonataCentral | 35 | 0.466 | 0.356 | −23.6% |
| *Ischnura cervula* | OdonataCentral | 66 | 0.601 | 0.687 | 14.3% |
| *Ischnura damula* | OdonataCentral | 60 | 0.226 | 0.287 | 27.0% |
| *Ischnura demorsa* | OdonataCentral | 31 | 0.133 | 0.239 | 80.3% |
| *Ischnura denticollis* | OdonataCentral | 49 | 0.344 | 0.313 | −9.2% |
| *Ischnura erratica* | OdonataCentral | 23 | 0.086 | 0.060 | −31.1% |
| *Ischnura hastata* | OdonataCentral | 240 | 0.574 | 0.591 | 2.9% |
| *Ischnura kellicotti* | OdonataCentral | 57 | 0.374 | 0.366 | −2.1% |
| *Ischnura perparva* | OdonataCentral | 73 | 0.570 | 0.695 | 21.9% |
| *Ischnura posita* | OdonataCentral | 395 | 0.552 | 0.584 | 5.9% |
| *Ischnura prognata* | OdonataCentral | 11 | 0.097 | 0.090 | −7.4% |
| *Ischnura ramburii* | OdonataCentral | 237 | 0.427 | 0.420 | −1.7% |
| *Ischnura verticalis* | OdonataCentral | 301 | 0.689 | 0.789 | 14.6% |
| *Ladona deplanata* | OdonataCentral | 167 | 0.423 | 0.437 | 3.3% |
| *Ladona exusta* | OdonataCentral | 11 | 0.049 | 0.040 | −19.3% |
| *Ladona julia* | OdonataCentral | 61 | 0.399 | 0.866 | 116.9% |
| *Lestes alacer* | OdonataCentral | 57 | 0.333 | 0.273 | −18.0% |
| *Lestes australis* | OdonataCentral | 187 | 0.529 | 0.604 | 14.2% |
| *Lestes congener* | OdonataCentral | 111 | 0.735 | 0.798 | 8.5% |
| *Lestes disjunctus* | OdonataCentral | 82 | 0.569 | 0.495 | −13.1% |
| *Lestes dryas* | OdonataCentral | 99 | 0.727 | 0.689 | −5.3% |
| *Lestes eurinus* | OdonataCentral | 34 | 0.312 | 0.375 | 20.2% |
| *Lestes forcipatus* | OdonataCentral | 49 | 0.647 | 0.738 | 14.1% |
| *Lestes forficula* | OdonataCentral | 14 | 0.251 | 0.269 | 7.3% |
| *Lestes inaequalis* | OdonataCentral | 69 | 0.401 | 0.434 | 8.3% |
| *Lestes rectangularis* | OdonataCentral | 171 | 0.550 | 0.624 | 13.5% |
| *Lestes sigma* | OdonataCentral | 12 | 0.046 | 0.032 | −31.9% |
| *Lestes unguiculatus* | OdonataCentral | 176 | 0.616 | 0.660 | 7.2% |
| *Lestes vigilax* | OdonataCentral | 88 | 0.391 | 0.393 | 0.5% |
| *Leucorrhinia borealis* | OdonataCentral | 12 | 0.126 | 0.082 | −34.4% |
| *Leucorrhinia frigida* | OdonataCentral | 31 | 0.179 | 0.166 | −7.6% |
| *Leucorrhinia glacialis* | OdonataCentral | 29 | 0.732 | 0.532 | −27.3% |
| *Leucorrhinia hudsonica* | OdonataCentral | 39 | 0.290 | 0.746 | 157.4% |
| *Leucorrhinia intacta* | OdonataCentral | 143 | 0.688 | 0.703 | 2.3% |
| *Leucorrhinia proxima* | OdonataCentral | 33 | 0.384 | 0.521 | 35.7% |
| *Libellula auripennis* | OdonataCentral | 58 | 0.400 | 0.401 | 0.4% |
| *Libellula axilena* | OdonataCentral | 49 | 0.235 | 0.250 | 6.3% |
| *Libellula comanche* | OdonataCentral | 53 | 0.230 | 0.531 | 131.2% |
| *Libellula composita* | OdonataCentral | 29 | 0.208 | 0.743 | 257.9% |
| *Libellula croceipennis* | OdonataCentral | 46 | 0.185 | 0.352 | 89.9% |
| *Libellula cyanea* | OdonataCentral | 163 | 0.370 | 0.354 | −4.3% |
| *Libellula flavida* | OdonataCentral | 54 | 0.279 | 0.266 | −4.6% |
| *Libellula forensis* | OdonataCentral | 114 | 0.439 | 0.388 | −11.7% |
| *Libellula incesta* | OdonataCentral | 304 | 0.483 | 0.509 | 5.3% |
| *Libellula luctuosa* | OdonataCentral | 497 | 0.846 | 0.915 | 8.1% |
| *Libellula needhami* | OdonataCentral | 63 | 0.261 | 0.243 | −6.8% |
| *Libellula nodisticta* | OdonataCentral | 26 | 0.234 | 0.364 | 55.4% |
| *Libellula pulchella* | OdonataCentral | 414 | 0.960 | 0.960 | 0.0% |
| *Libellula quadrimaculata* | OdonataCentral | 148 | 0.562 | 0.615 | 9.5% |
| *Libellula saturata* | OdonataCentral | 136 | 0.478 | 0.669 | 40.0% |
| *Libellula semifasciata* | OdonataCentral | 69 | 0.378 | 0.365 | −3.4% |
| *Libellula vibrans* | OdonataCentral | 231 | 0.542 | 0.562 | 3.6% |
| *Macrodiplax balteata* | OdonataCentral | 53 | 0.210 | 0.837 | 298.1% |
| *Macromia annulata* | OdonataCentral | 15 | 0.047 | 0.117 | 148.8% |
| *Macromia illinoiensis* | OdonataCentral | 62 | 0.528 | 0.521 | −1.4% |
| *Macromia magnifica* | OdonataCentral | 21 | 0.285 | 0.385 | 35.1% |
| *Macromia pacifica* | OdonataCentral | 13 | 0.509 | 0.547 | 7.4% |
| *Macromia taeniolata* | OdonataCentral | 61 | 0.425 | 0.411 | −3.4% |
| *Miathyria marcella* | OdonataCentral | 43 | 0.347 | 0.408 | 17.6% |
| *Micrathyria hagenii* | OdonataCentral | 23 | 0.136 | 0.047 | −65.3% |
| *Nannothemis bella* | OdonataCentral | 19 | 0.366 | 0.328 | −10.3% |
| *Nasiaeschna pentacantha* | OdonataCentral | 83 | 0.540 | 0.523 | −3.0% |
| *Nehalennia gracilis* | OdonataCentral | 29 | 0.245 | 0.200 | −18.5% |
| *Nehalennia integricollis* | OdonataCentral | 35 | 0.219 | 0.188 | −14.5% |
| *Nehalennia irene* | OdonataCentral | 65 | 0.436 | 0.704 | 61.5% |
| *Neoneura aaroni* | OdonataCentral | 13 | 0.052 | 0.044 | −14.9% |
| *Neurocordulia molesta* | OdonataCentral | 15 | 0.335 | 0.322 | −3.8% |
| *Neurocordulia xanthosoma* | OdonataCentral | 20 | 0.074 | 0.134 | 79.9% |
| *Neurocordulia yamaskanensis* | OdonataCentral | 13 | 0.285 | 0.280 | −1.9% |
| *Octogomphus specularis* | OdonataCentral | 12 | 0.079 | 0.091 | 14.5% |
| *Ophiogomphus occidentis* | OdonataCentral | 16 | 0.121 | 0.153 | 26.6% |
| *Ophiogomphus rupinsulensis* | OdonataCentral | 21 | 0.236 | 0.297 | 25.8% |
| *Ophiogomphus severus* | OdonataCentral | 77 | 0.518 | 0.462 | −10.9% |
| *Orthemis discolor* | OdonataCentral | 28 | 0.157 | 0.173 | 10.0% |
| *Orthemis ferruginea* | OdonataCentral | 168 | 0.447 | 0.548 | 22.4% |
| *Pachydiplax longipennis* | OdonataCentral | 619 | 0.965 | 0.884 | −8.4% |
| *Paltothemis lineatipes* | OdonataCentral | 25 | 0.197 | 0.213 | 8.3% |
| *Pantala flavescens* | OdonataCentral | 246 | 0.763 | 0.897 | 17.5% |
| *Pantala hymenaea* | OdonataCentral | 199 | 0.819 | 0.907 | 10.7% |
| *Perithemis domitia* | OdonataCentral | 16 | 0.041 | 0.057 | 40.1% |
| *Perithemis tenera* | OdonataCentral | 497 | 0.916 | 0.861 | −6.0% |
| *Phanogomphus descriptus* | OdonataCentral | 11 | 0.061 | 0.071 | 15.70% |
| *Phanogomphus exilis* | OdonataCentral | 107 | 0.373 | 0.342 | −8.1% |
| *Phanogomphus graslinellus* | OdonataCentral | 47 | 0.735 | 0.697 | −5.2% |
| *Phanogomphus kurilis* | OdonataCentral | 13 | 0.057 | 0.053 | −6.8% |
| *Phanogomphus lividus* | OdonataCentral | 76 | 0.335 | 0.342 | 1.90% |
| *Phanogomphus militaris* | OdonataCentral | 119 | 0.335 | 0.449 | 34.00% |
| *Phanogomphus oklahomensis* | OdonataCentral | 35 | 0.186 | 0.148 | −20.4% |
| *Phanogomphus quadricolor* | OdonataCentral | 19 | 0.287 | 0.262 | −8.9% |
| *Phanogomphus spicatus* | OdonataCentral | 19 | 0.165 | 0.146 | −11.2% |
| *Phyllogomphoides albrighti* | OdonataCentral | 23 | 0.029 | 0.031 | 7.3% |
| *Phyllogomphoides stigmatus* | OdonataCentral | 48 | 0.126 | 0.138 | 10.0% |
| *Plathemis lydia* | OdonataCentral | 571 | 0.944 | 0.997 | 5.6% |
| *Plathemis subornata* | OdonataCentral | 36 | 0.288 | 0.388 | 34.7% |
| *Progomphus borealis* | OdonataCentral | 23 | 0.176 | 0.334 | 89.4% |
| *Progomphus obscurus* | OdonataCentral | 135 | 0.364 | 0.420 | 15.6% |
| *Protoneura cara* | OdonataCentral | 14 | 0.050 | 0.060 | 20.4% |
| *Pseudoleon superbus* | OdonataCentral | 30 | 0.142 | 0.160 | 12.9% |
| *Rhionaeschna californica* | OdonataCentral | 69 | 0.388 | 0.555 | 42.8% |
| *Rhionaeschna multicolor* | OdonataCentral | 220 | 0.960 | 0.842 | −12.3% |
| *Rhionaeschna mutata* | OdonataCentral | 22 | 0.095 | 0.123 | 28.9% |
| *Rhionaeschna psilus* | OdonataCentral | 12 | 0.300 | 0.258 | −14.1% |
| *Somatochlora albicincta* | OdonataCentral | 18 | 0.052 | 0.147 | 183.9% |
| *Somatochlora ensigera* | OdonataCentral | 17 | 0.226 | 0.205 | −9.2% |
| *Somatochlora filosa* | OdonataCentral | 10 | 0.223 | 0.203 | −8.8% |
| *Somatochlora linearis* | OdonataCentral | 53 | 0.342 | 0.352 | 2.8% |
| *Somatochlora minor* | OdonataCentral | 12 | 0.147 | 0.331 | 125.8% |
| *Somatochlora semicircularis* | OdonataCentral | 55 | 0.110 | 0.303 | 175.5% |
| *Somatochlora tenebrosa* | OdonataCentral | 37 | 0.209 | 0.216 | 3.1% |
| *Somatochlora walshii* | OdonataCentral | 27 | 0.136 | 0.355 | 160.6% |
| *Somatochlora williamsoni* | OdonataCentral | 10 | 0.080 | 0.091 | 13.7% |
| *Stenogomphus rogersi* | OdonataCentral | 12 | 0.066 | 0.075 | 12.30% |
| *Stylogomphus albistylus* | OdonataCentral | 23 | 0.262 | 0.271 | 3.2% |
| *Stylurus intricatus* | OdonataCentral | 14 | 0.338 | 0.485 | 43.5% |
| *Stylurus laurae* | OdonataCentral | 11 | 0.148 | 0.094 | −36.2% |
| *Stylurus olivaceus* | OdonataCentral | 23 | 0.240 | 0.204 | −15.3% |
| *Stylurus plagiatus* | OdonataCentral | 98 | 0.363 | 0.627 | 72.9% |
| *Sympetrum ambiguum* | OdonataCentral | 101 | 0.665 | 0.663 | −0.4% |
| *Sympetrum corruptum* | OdonataCentral | 394 | 0.989 | 0.925 | −6.5% |
| *Sympetrum costiferum* | OdonataCentral | 109 | 0.451 | 0.519 | 15.3% |
| *Sympetrum danae* | OdonataCentral | 89 | 0.442 | 0.543 | 22.9% |
| *Sympetrum illotum* | OdonataCentral | 38 | 0.684 | 0.462 | −32.5% |
| *Sympetrum internum* | OdonataCentral | 127 | 0.673 | 0.578 | −14.1% |
| *Sympetrum madidum* | OdonataCentral | 53 | 0.436 | 0.389 | −10.9% |
| *Sympetrum obtrusum* | OdonataCentral | 191 | 0.654 | 0.736 | 12.5% |
| *Sympetrum pallipes* | OdonataCentral | 143 | 0.583 | 0.471 | −19.4% |
| *Sympetrum rubicundulum* | OdonataCentral | 77 | 0.521 | 0.513 | −1.4% |
| *Sympetrum semicinctum* | OdonataCentral | 227 | 0.886 | 0.907 | 2.4% |
| *Sympetrum vicinum* | OdonataCentral | 208 | 0.823 | 0.941 | 14.3% |
| *Tachopteryx thoreyi* | OdonataCentral | 49 | 0.312 | 0.298 | −4.4% |
| *Tanypteryx hageni* | OdonataCentral | 21 | 0.038 | 0.073 | 93.2% |
| *Telebasis byersi* | OdonataCentral | 34 | 0.198 | 0.192 | −3.1% |
| *Telebasis salva* | OdonataCentral | 80 | 0.217 | 0.211 | −3.0% |
| *Tramea calverti* | OdonataCentral | 40 | 0.492 | 0.552 | 12.0% |
| *Tramea carolina* | OdonataCentral | 101 | 0.322 | 0.342 | 6.1% |
| *Tramea lacerata* | OdonataCentral | 387 | 0.920 | 0.943 | 2.5% |
| *Tramea onusta* | OdonataCentral | 233 | 0.622 | 0.926 | 48.9% |
| *Triacanthagyna trifida* | OdonataCentral | 11 | 0.027 | 0.028 | 2.2% |
